# Improving the Annotation Process in Computational Pathology: A Pilot Study with Manual and Semi-automated Approaches on Consumer and Medical Grade Devices

**DOI:** 10.1007/s10278-024-01248-x

**Published:** 2024-09-04

**Authors:** Giorgio Cazzaniga, Fabio Del Carro, Albino Eccher, Jan Ulrich Becker, Giovanni Gambaro, Mattia Rossi, Federico Pieruzzi, Filippo Fraggetta, Fabio Pagni, Vincenzo L’Imperio

**Affiliations:** 1 Department of Medicine and Surgery, Pathology, IRCCS Fondazione San Gerardo Dei Tintori, University of Milano-Bicocca, Via Pergolesi, 33, 20900 Monza, Italy; 2 Department of Medical and Surgical Sciences for Children and Adults, University of Modena and Reggio Emilia, University Hospital of Modena, Modena, Italy; 3 Institute of Pathology, University Hospital of Cologne, Cologne, Germany; 4 Division of Nephrology, Department of Medicine, University of Verona, Verona, Italy; 5 Clinical Nephrology, Fondazione IRCCS San Gerardo Dei Tintori, Monza, Italy; 6 School of Medicine and Surgery, University of Milano-Bicocca, Milan, Italy; 7 Pathology Unit, Azienda Sanitaria Provinciale (ASP) Catania, “Gravina” Hospital, Caltagirone, Italy

**Keywords:** Annotation, Digital pathology, Artificial intelligence, Segment Anything Model, Computational pathology

## Abstract

The development of reliable artificial intelligence (AI) algorithms in pathology often depends on ground truth provided by annotation of whole slide images (WSI), a time-consuming and operator-dependent process. A comparative analysis of different annotation approaches is performed to streamline this process. Two pathologists annotated renal tissue using semi-automated (Segment Anything Model, SAM)) and manual devices (touchpad vs mouse). A comparison was conducted in terms of working time, reproducibility (overlap fraction), and precision (0 to 10 accuracy rated by two expert nephropathologists) among different methods and operators. The impact of different displays on mouse performance was evaluated. Annotations focused on three tissue compartments: tubules (57 annotations), glomeruli (53 annotations), and arteries (58 annotations). The semi-automatic approach was the fastest and had the least inter-observer variability, averaging 13.6 ± 0.2 min with a difference (*Δ*) of 2%, followed by the mouse (29.9 ± 10.2, *Δ* = 24%), and the touchpad (47.5 ± 19.6 min, *Δ* = 45%). The highest reproducibility in tubules and glomeruli was achieved with SAM (overlap values of 1 and 0.99 compared to 0.97 for the mouse and 0.94 and 0.93 for the touchpad), though SAM had lower reproducibility in arteries (overlap value of 0.89 compared to 0.94 for both the mouse and touchpad). No precision differences were observed between operators (*p* = 0.59). Using non-medical monitors increased annotation times by 6.1%. The future employment of semi-automated and AI-assisted approaches can significantly speed up the annotation process, improving the ground truth for AI tool development.

**Supplementary Information:**

The online version contains supplementary material available at 10.1007/s10278-024-01248-x.

## Introduction

The introduction of artificial intelligence (AI) algorithms in pathology promises to increase diagnostic efficiency, accelerating the analysis of pathological images [1–3], alleviating the burden of repetitive and error-prone tasks, and optimizing the accuracy and speed of pathological evaluations [4]. Achieving a robust ground truth for supervised AI models often involves a meticulous whole slide image (WSI) annotation process, with pathologists contributing their expertise in tissue interpretation to the model construction [5, 6]. This process is meant to delineate regions of interest (ROIs), facilitating model training for accurate detection and segmentation [7]. However, the annotation process is time-consuming, laborious, and operator-dependent, whose inter-observer variability can potentially hamper the construction of a reliable dataset, jeopardizing generalizability [8, 9]. Moreover, depending on the desired task (e.g., classification, detection, semantic segmentation, or instance segmentation), different types of annotation may be required, with harmonization efforts in this setting still lagging back [10, 11]. Finally, different equipment and devices are becoming available on the market with the promise to improve the annotation process, but traditional mouse and keyboard on commercially available and non-medical monitors still remain the most widespread approach [12, 13]. Manual annotation involves experts using various devices, such as mouse or touchpads, each with different interaction styles, grips, and movements, to meticulously label images. While this method ensures precise control, it is labor-intensive and time-consuming. Semi-automatic approaches enhance this process by combining initial manual annotations with automated tools. Techniques like Active Learning and the Segment Anything Model (SAM) streamline the workflow, improving efficiency and reducing time spent on manual labeling [14]. Recently, automatic annotation methods have emerged, leveraging advanced AI, particularly unsupervised techniques at the patch level, to fully automate the labeling process, offering rapid and consistent results with minimal human intervention [15]. Here, a benchmark of manual (mouse vs touchpad) and semi-automated (AI-assisted) annotation methods is performed [16], comparing also the performances on medical and consumer displays to provide insights on the impact of these combinations in the peculiar nephropathology niche.

## Methods

### Annotation Process

**Fig. 1:**
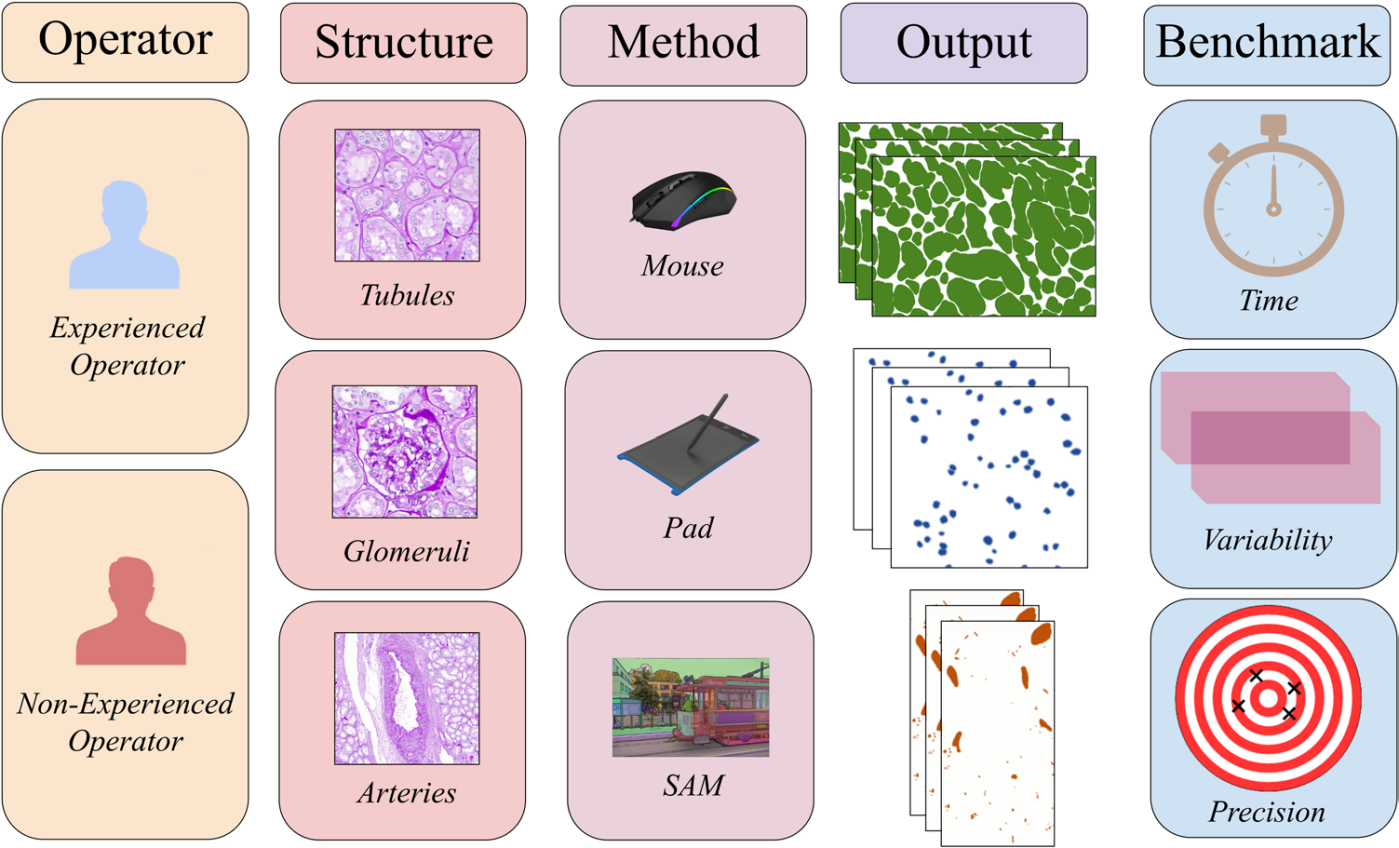
Graphic representation of the study design. Two pathologists, one with experience in annotation and the other at its beginner phase with this task, were asked to annotate three different renal structures (tubules, glomeruli, and arteries) using two manual devices (mouse vs touchpad) and a semi-automatic, AI-based tool (SAM). Starting from the masks obtained with this annotation process, performances in terms of working time required, inter-observer variability, and annotation precision were calculated, comparing the results obtained with the mouse using medical and non-medical displays

**Fig. 2:**
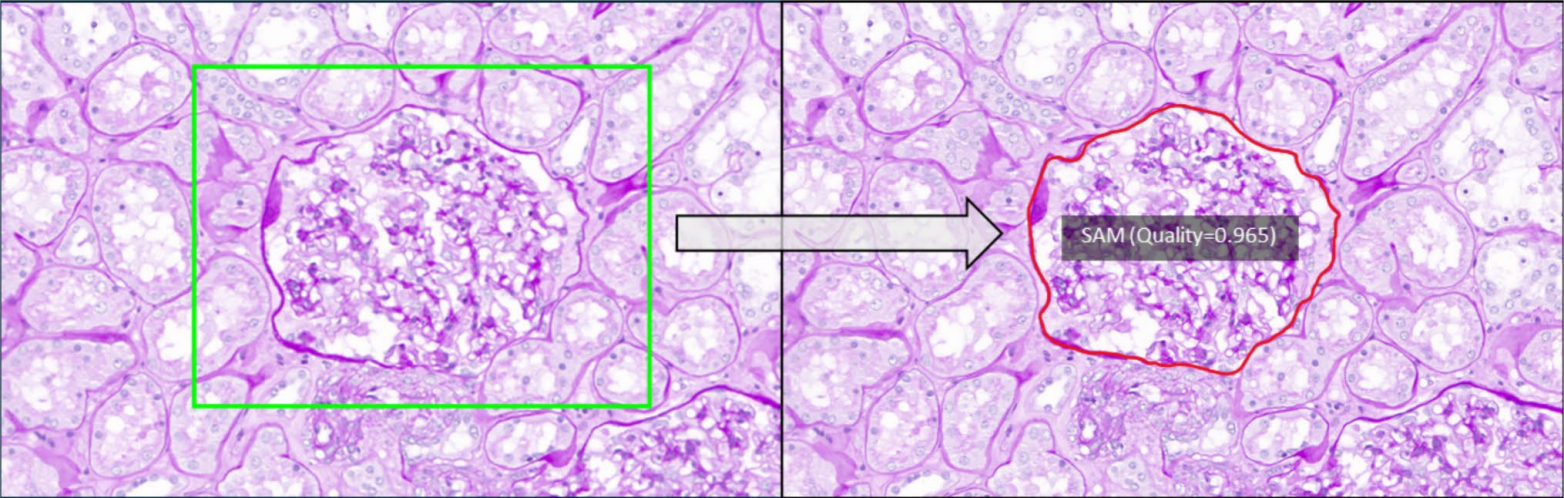
The Qupath extension for SAM allows the user to draw a bounding box around the structure of interest and let the model finely annotate it

A schematic representation of the study design is reported in Fig. 1. Two different pathologists, one with a 5-year experience in the annotation practice in nephropathology (VL) and a 2nd-year pathology resident (FDC, Supplementary Material), were asked to annotate tubules, glomeruli, and arteries from a single PAS-stained WSI of renal cortex extracted from the Digital Nephropathology Archives of Fondazione IRCCS San Gerardo dei Tintori, University of Milano-Bicocca, Monza, Italy [17]. Each operator was asked to replicate the annotations on each ROI based on a predefined ground truth, using two manual methods, the traditional mouse (Fujitsu M-U0026, Tokyo, Japan) vs touchpad (integrated within the used monitor BARCO MDPC-8127, Courtrai, Belgium), and a semi-automatic/AI-assisted method (SAM, https://github.com/ksugar/qupath-extension-sam, v0.4.1) [18–20], using the WSI viewing and annotation software QuPath (v0.4.4, Supplementary Material) [21]. This latter semi-automatic method requires that the annotator recognizes roughly the structure of interest (e.g., glomerulus), including it in a square/rectangle allowing the algorithm to finely outline the structure itself. For the first ROI containing tubules, the “Auto mask” tool was preferred, whereas for the second and third ROIs, which included glomeruli and arteries, the operators favored using a prompt with the rectangle tool for bounding box annotations (Fig. 2). Annotations were conducted over three separate sessions, with a 2-week washout period between each. The sessions included the use of a mouse, a touchpad, and SAM in a sequential order for both operators. Each operator had the opportunity to familiarize with the touchpad and the SAM before commencing the experiment for an adequate period of training.

The ground truth was established by an expert nephropathologist (JUB) who pointed out the presence of each structure (glomeruli, tubules, and arteries) in three different regions of the slide. Annotations were performed on a pathology-dedicated medical workstation with a BARCO MDPC-8127 monitor (27″ diagonal, 3840 × 2160 pixels, 120 Hz refresh rate). For comparison, the same operators also performed mouse-based annotations on a consumer off-the-shelf workstation with an HANNS-G HP205 monitor (19″ diagonal, 1600 × 900 pixels 60 Hz refresh rate).

### Statistical Analysis

The time required to annotate each type of structure and the annotation method used were recorded from the beginning to the end of the annotation process for each group of objects. The average annotation time across the two annotators was reported as mean ± standard deviation (sd) and as median and interquartile range, while time variability was calculated as the percentage difference between two values (*Δ***)**. To assess the inter-observer and inter-method annotation reproducibility, a binary mask was extracted from the obtained annotations. Traditional metrics like Intersection over Union (IoU) may be prone to over/underestimation of results due to background interferences (the larger the background area and the smaller the annotated area, the better the IoU, assuming annotation correctness remains constant). To mitigate this, we employed a simpler measure called the overlap fraction (*ov*):

$$\text{overlap fraction }\left(ov\right)=\frac{\text{overlap annotated pixels}}{\text{total annotated pixels}}$$

This metric focuses solely on annotations without considering background interference, enabling a more comparable precision evaluation across various anatomical structures (Supplementary Material). Moreover, the obtained annotations were evaluated for accuracy by two expert nephropathologists (AE and FP), asked to provide a semi-quantitative score to the quality of annotations after unanimous agreement, ranging from 0 to 10. The non-parametric test (Mann–Whitney *U* test) was used across all times and scores reached by the two annotators to assess the disparities in annotation accuracy, considering *p*-values < 0.05 as statistically significant. All the data extracted were subjected to statistical analysis using Python (v3.10) libraries such as pandas (v2.2) and scikit-learn (v1.1).

Spreadsheets are deposited in Bicocca Open Archive Research Data (BOARD): Cazzaniga, Giorgio; L’Imperio, Vincenzo (2024), “Improving the annotation process in computational pathology: from manual to semi-automatic approaches in digital nephropathology,” Bicocca Open Archive Research Data, V1, doi: 10.17632/c36ywkzrm9.1. This paper does not report original code.

## Results

### Annotation Time Variability, Reproducibility, and Accuracy

**Fig. 3:**
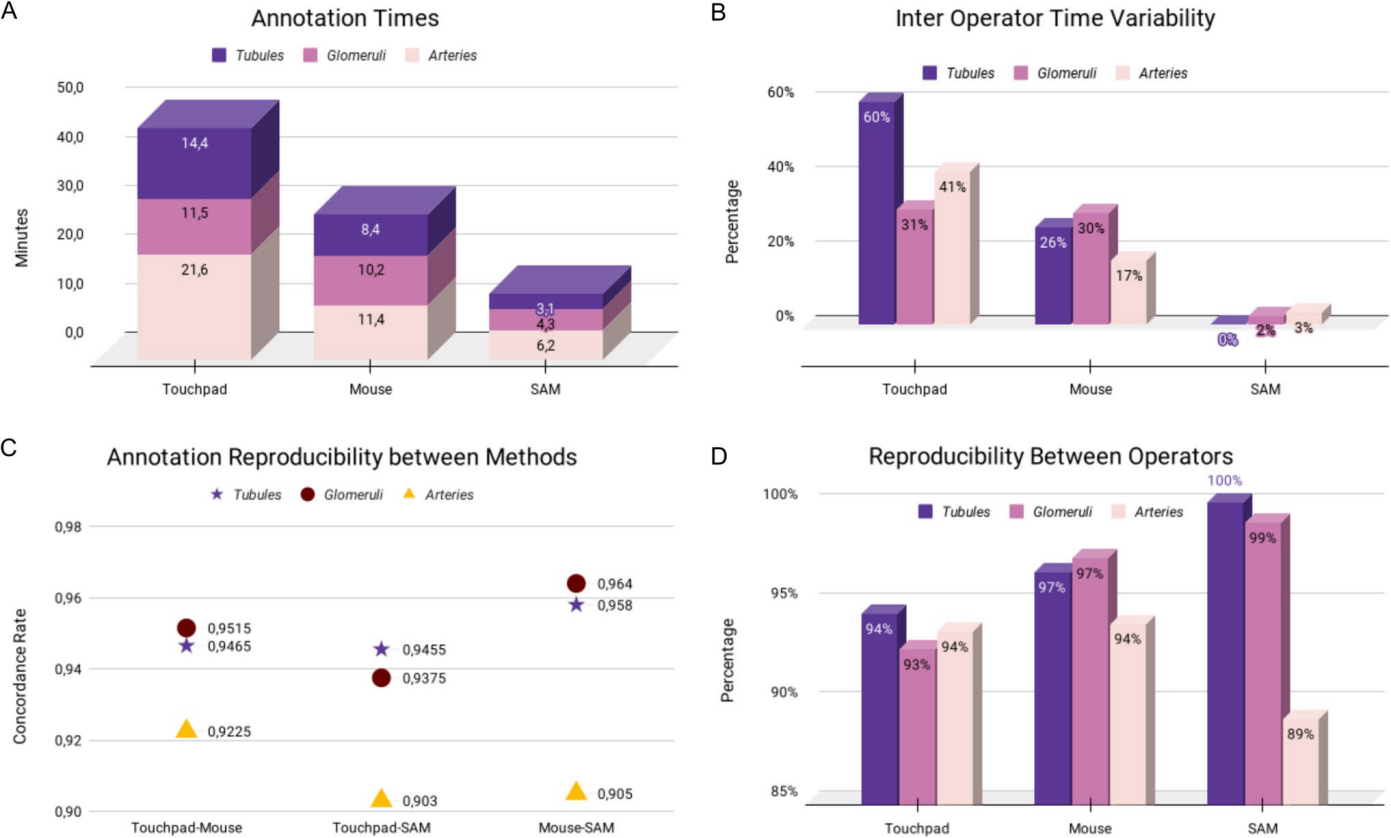
The inter-annotator average times required for the annotation of different structures (tubules, violet; glomeruli, pink; arteries, light pink) using different devices (touchpad, mouse, and SAM) (**A**). Time variability between the two annotators is reported in %, using the same stratification (**B**). Finally, the reproducibility in terms of overlap fraction for ROIs obtained with different methods (**C**) and from the two observers (**D**) is reported

A total of 57 tubules, 53 glomeruli, and 58 arteries were annotated by both the annotators with all the methods, accounting for 168 structures. SAM was the fastest, with an inter-annotator average time of 13.6 ± 0.2 min (median 4.3 min, IQR 3.38–5.65) and the lowest time variability between annotators (*Δ* = 2%, Fig. 3A, B). This was followed by the traditional mouse method, which had an average time of 29.9 ± 10.2 min (median 10 min, IQR 7.68–12.55, *Δ* = 24%), and the touchpad method, with an average time of 47.5 ± 19.6 min (median 14.8 min, IQR 10.45–19.38, *Δ* = 45%). Comparing the methods, the touchpad was 59% slower than the mouse, while the manual methods were significantly slower than the semi-automatic approach, with the mouse and the touchpad being 121% and 249% slower than SAM, respectively. The differences in annotation time were statistically significant when comparing SAM to both the mouse and touchpad (*p* < 0.01), but not significant when comparing the mouse to the touchpad (*p* = 0.11). Overall, the time differences between the two annotators were not statistically significant (*p* = 0.27).

Overall, SAM vs mouse demonstrated the best annotation reproducibility assessed through the annotation overlap (*ov* = 0.96), with best performances for tubules and glomeruli, as compared to the slightly poorer touchpad performances (*ov* = 0.94 and 0.93 vs mouse and SAM, respectively, Fig. 3C). Similar superiority was noted for inter-observer reproducibility for semi-automated (*ov* = 0.96) vs manual (*ov* = 0.96 and 0.94 for mouse and touchpad, respectively), although SAM showed the worst performances in arteries annotation (*ov* = 0.89, Fig. 3D). Overall, a statistically significant difference was observed in the annotation reproducibility between the two operators when considering all methods together (*p* = 0.03).

**Fig. 4:**
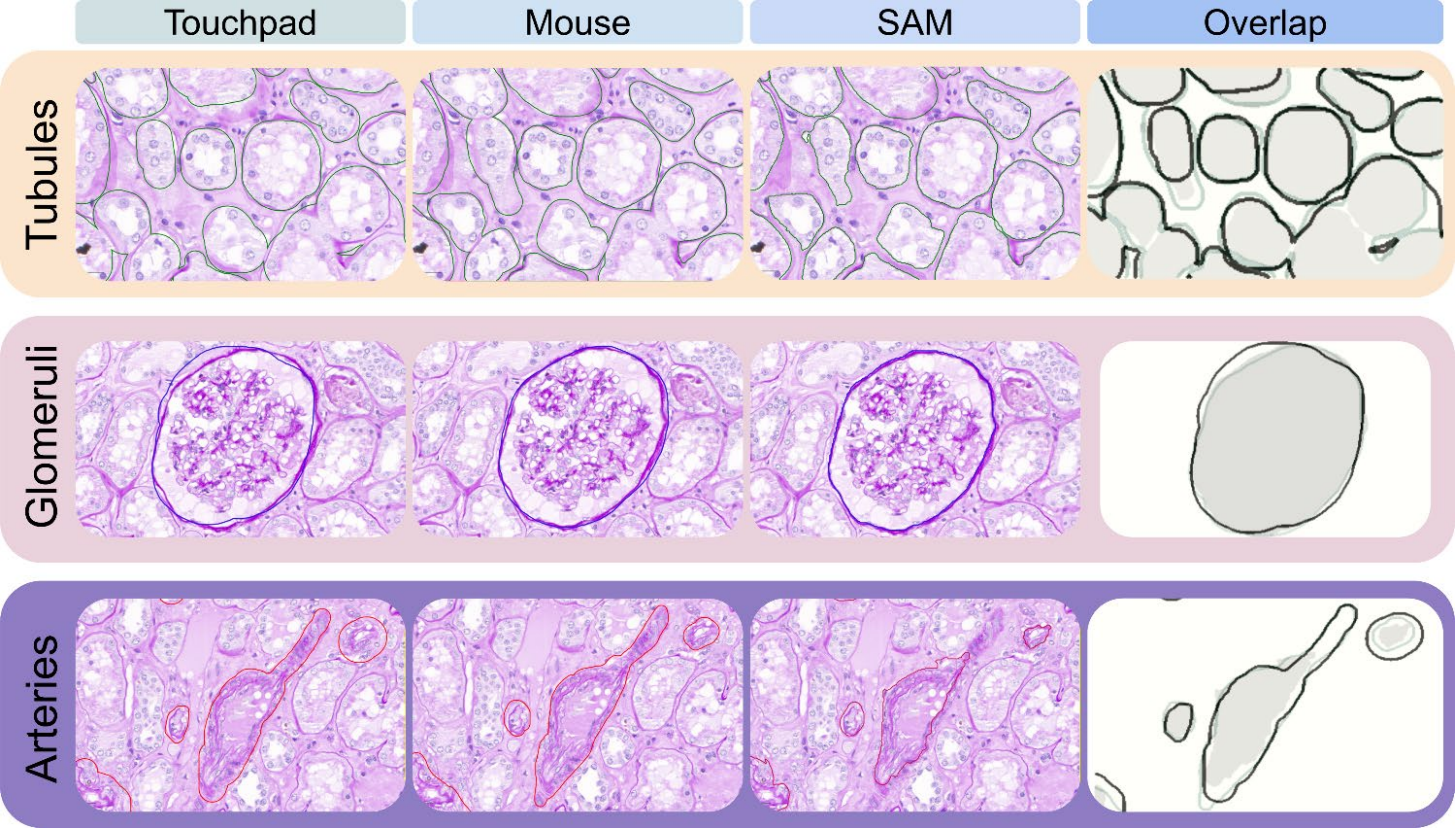
Comparison of the annotation outlines for tubules, glomeruli, and arteries obtained with the different devices (touchpad, mouse, and SAM), with relative overlap of the ROIs obtained

The accuracy scores attributed to annotations from the two expert nephropathologists are reported in Table 1. This analysis confirmed the superiority of SAM in annotating tubules and glomeruli, with the manual methods outperforming on arteries (Fig. 4). No significant differences in terms of accuracy were noted between the two annotators (*p* = 1) in this setting.

**Table 1:** Accuracy scores assigned by expert nephropathologists to annotations, categorized by annotator (#1 and #2) and annotation method

	**A1 pad**	**A1 mouse**	**A1 SAM**	**A2 pad**	**A2 mouse**	**A2 SAM**
Tubules	7	7	10	6	8	10
Glomeruli	7	8	10	7	8	10
Arteries	7	9	5	7	8	5
Ov. mean	7	8	8.33	6.67	8	8.33
Ov. median	7	8	10	7	8	10

### Impact of Displays on the Annotation Process

The comparative analysis of annotations obtained with mouse on pathology-dedicated, medical, and consumer/non-medical monitors did not show substantial differences in terms of annotation accuracy. Excellent inter-observer reproducibility was noted on the consumer monitor (*ov* = 0.95, 0.98, and 0.95 for tubules, glomeruli, and arteries, respectively), and the same result was achieved comparing annotations obtained with the two monitors, independently from the operator (0.95, 0.96, and 0.94, respectively). The annotation process on the consumer monitor resulted in an overall increase in annotation time by 6.1%, with varying impacts on different renal structures: tubules increased by 5.1%, glomeruli by 4.7%, and arteries by 8.1% (Table 2). However, these differences were overall not statistically significant (*p* = 0.589).

**Table 2:** Time comparison between the medical and the consumer monitor (minutes). A1 and A2, annotator #1 and #2

	**Medical monitor**			**Consumer monitor**			**Mean *Δ***
	**A1**	**A2**	**Mean**	**A1**	**A2**	**Mean**	
**Tubules**	6.2	10.6	8.4	6.9	10.8	8.9	5.1%
**Glomeruli**	7.1	13.2	10.15	7.3	14	10.7	4.7%
**Arteries**	9.4	13.3	11.35	10	14.7	12.4	8.1%
**Overall**	22.7	37.1	29.9	24.2	39.5	31.9	6.1%

## Discussion

The increasing interest in developing and implementing AI algorithms in pathology encounters various challenges [22, 23]. These include acquiring large volumes of whole slide images (WSIs) for training, which is hindered by the incomplete digitization of routine pathology practices [24, 25]. Additionally, there is a demand for specialized expertise in identifying regions of interest (ROI) and recognizing specific histological lesions, particularly in niche pathology areas [26, 27]. These criticalities are stimulating the establishment of collaborative networks, with dedicated personnel accounting for the annotation of large datasets for particular tasks [28], emphasizing the importance of adopting a careful, step-by-step strategy [29–32]. However, precise annotations are crucial to guarantee the reproducibility and generalizability of the results, and this process is still the time-limiting step for AI algorithm development, with lacking internationally recognized guidelines. In this setting, detecting cost and time-effective alternative methods can potentially boost the development of supervised AI algorithms [33], potentially impacting the final outcome of the model [23, 34, 35]. The present study investigates the introduction of technological novelties in the pathology workstation and its impact on time and accuracy of the annotation process [36]. In particular, the conventional mouse with keyboard shortcuts was compared to a monitor-integrated touchpad with customizable buttons (e.g., zooming, navigating, and selecting) [37] and to semi-automatic approaches based on SAM [38]. The latter method showed superior performances in terms of annotation time and time variability between annotators, suggesting possible minimization of the training phase for beginners, just requiring a final rapid review of automatically recognized structures to avoid non-interesting regions or artifacts [39]. Similarly, the SAM approach showed superior reproducibility independently from the annotator, especially for tubule identification, with slightly worse performances on glomeruli and especially arteries. This would imply a standardization of the different structure annotations that can prospectively guarantee the harmonization and generalizability of the deriving AI algorithms. On the other hand, as expected, manual methods proved to be more time-consuming, with the mouse demonstrating slightly faster results compared to the touchpad. This difference may be attributed to the touchpad’s design and optimization for navigation rather than annotation. Independently from the annotation device used, more sharply defined structures (e.g., tubules/glomeruli) were characterized by a more precise and fast annotation, as reflected by the overall lower performances noted with arteries in this study. In this setting, the pathologist’s experience plays a crucial role, elevating manual annotations to higher levels of reproducibility and precision compared to semi-automatic methods, as confirmed by the accuracy scores attributed by expert nephropathologists. We acknowledge some limitations of the study, which may hamper the generalizability of the results, including a limited number of subjects and the use of a single image. However, the selected subfield presents a valuable array of structures that make it an ideal benchmark for pathology annotations. It includes easily identifiable, densely packed structures like tubules, scattered elements such as glomeruli, and more intricate formations with less defined boundaries, such as arteries, making it a great benchmark for annotations in pathology. Although there are benefits, the employment of semi-automated and AI-based annotation tools is still lagging behind, probably due to the steeper learning curve and complexity of the approach for general pathologists as compared to the daily-used mouse or touchpad [7]. In addition to the findings presented, it is worth noting that models specifically tailored to particular domains, as opposed to general ones, may offer enhanced performance, such as the ones trained specifically with pathology images [40]. At the same time, the rapid and increasing development of AI models that allow for partial annotation of a dataset, enabling the model to learn in real-time, is demonstrating significant benefits in annotating large volumes of images. This approach facilitates automation that alleviates the manual task of annotating structures and ensures a streamlined process [41, 42]. Efforts should be made to simplify the access to these tools, democratizing these instruments to the general pathology public to boost the development of useful AI algorithms for the clinical use in sub-specialized niches (e.g., nephropathology [43]). Finally, although the use of a medical grade monitor allowed a smoother navigation of the virtual slide, these results highlight the importance of the selection of the most appropriate annotation tool across different monitors, stressing that even marginal enhancements in the technical setup can contribute to more efficient workflow [44–46].

## Conclusion

The future employment of semi-automated and AI-assisted approaches can significantly speed up the annotation process, improving the ground truth for AI tool development and testing and reducing inter-observer variability. The implementation of new technological instrumentation can further enrich the digital pathology capabilities of the lab. The democratization of these innovations can contribute to reducing the reluctance on the adoption of digital pathology transition in our labs. 

## Supplementary Information

Below is the link to the electronic supplementary material.

Supplementary file1 : Examples of annotations with high and low accuracy. In the upper row, the Bowman’s capsule and the basal membrane of the tubules are followed precisely, with no inclusion of interstitial tissue nor exclusion of areas that are part of the structures of interest; the artery is fully included, encompassing all layers: intima, media, and adventitia. In the lower row, however, the membranes are not followed precisely, and only the intima layer and the lumen of the vessel are included, as SAM has identified the boundaries with high contrast of the elastic lamina compared to the more blurred contours between the adventitia and the interstitium (PNG 4047 KB)

Supplementary file2 (DOCX 9 KB)

## Data Availability

Spreadsheets are deposited in Bicocca Open Archive Research Data (BOARD): Cazzaniga, Giorgio; L’Imperio, Vincenzo (2024), “Improving the annotation process in computational pathology: from manual to semi-automatic approaches in digital nephropathology,” Bicocca Open Archive Research Data, V1, doi: 10.17632/c36ywkzrm9.1. This paper does not report original code.

## References

[1] Hanna, M. G., Ardon, O., Reuter, V. E., Sirintrapun, S. J., England, C., Klimstra, D. S. & Hameed, M. R. Integrating digital pathology into clinical practice. *Mod. Pathol.* **35,** 152–164 (2022). 34599281 10.1038/s41379-021-00929-0

[2] Pisapia, P., L’Imperio, V., Galuppini, F., Sajjadi, E., Russo, A., Cerbelli, B., Fraggetta, F., d’Amati, G., Troncone, G., Fassan, M., Fusco, N., Pagni, F. & Malapelle, U. The evolving landscape of anatomic pathology. *Crit. Rev. Oncol. Hematol.* **178,** 103776 (2022). 35934262 10.1016/j.critrevonc.2022.103776

[3] L’Imperio, V., Casati, G., Cazzaniga, G., Tarabini, A., Bolognesi, M. M., Gibilisco, F., Fraggetta, F. & Pagni, F. Improvements in digital pathology equipment for renal biopsies: updating the standard model. *J. Nephrol.* (2023). 10.1007/s40620-023-01568-1 36786977 10.1007/s40620-023-01568-1

[4] Cazzaniga, G., Rossi, M., Eccher, A., Girolami, I., L’Imperio, V., Van Nguyen, H., Becker, J. U., Bueno García, M. G., Sbaraglia, M., Dei Tos, A. P., Gambaro, G. & Pagni, F. Time for a full digital approach in nephropathology: a systematic review of current artificial intelligence applications and future directions. *J. Nephrol.* (2023). 10.1007/s40620-023-01775-w 37768550 10.1007/s40620-023-01775-w PMC10920416

[5] Niazi, M. K. K., Parwani, A. V. & Gurcan, M. N. Digital pathology and artificial intelligence. *Lancet Oncol.* **20,** e253–e261 (2019). 31044723 10.1016/S1470-2045(19)30154-8 PMC8711251

[6] Ball, E., Franks, H., Jenkins, J., McGrath, M. & Leigh, J. Annotation is a valuable tool to enhance learning and assessment in student essays. *Nurse Educ. Today* **29,** 284–291 (2009). 19084297 10.1016/j.nedt.2008.10.005

[7] Montezuma, D., Oliveira, S. P., Neto, P. C., Oliveira, D., Monteiro, A., Cardoso, J. S. & Macedo-Pinto, I. Annotating for Artificial Intelligence Applications in Digital Pathology: A Practical Guide for Pathologists and Researchers. *Mod. Pathol.* **36,** 100086 (2023). 36788085 10.1016/j.modpat.2022.100086

[8] Pati, P., Foncubierta-Rodríguez, A., Goksel, O. & Gabrani, M. Reducing annotation effort in digital pathology: A Co-Representation learning framework for classification tasks. *Med. Image Anal.* **67,** 101859 (2021). 33129150 10.1016/j.media.2020.101859

[9] Foucart, A., Debeir, O. & Decaestecker, C. Shortcomings and areas for improvement in digital pathology image segmentation challenges. *Comput. Med. Imaging Graph.* **103,** 102155 (2023). 36525770 10.1016/j.compmedimag.2022.102155

[10] Li, B., Mercan, E., Mehta, S., Knezevich, S., Arnold, C. W., Weaver, D. L., Elmore, J. G. & Shapiro, L. G. Classifying Breast Histopathology Images with a Ductal Instance-Oriented Pipeline. *Proc. IAPR Int. Conf. Pattern Recogn.* **2020,** 8727–8734 (2021). 36745147 10.1109/icpr48806.2021.9412824 PMC9893896

[11] Deng, S., Zhang, X., Yan, W., Chang, E. I.-C., Fan, Y., Lai, M. & Xu, Y. Deep learning in digital pathology image analysis: a survey. *Front. Med.* **14,** 470–487 (2020). 32728875 10.1007/s11684-020-0782-9

[12] Evans, H., Hero, E., Minhas, F., Wahab, N., Dodd, K., Sahota, H., Ganguly, R., Robinson, A., Neerudu, M., Blessing, E., Borkar, P. & Snead, D. Standardized Clinical Annotation of Digital Histopathology Slides at the Point of Diagnosis. *Mod. Pathol.* **36,** 100297 (2023). 37544362 10.1016/j.modpat.2023.100297

[13] Marée, R. Open Practices and Resources for Collaborative Digital Pathology. *Front. Med.* **6,** 255 (2019). 10.3389/fmed.2019.00255 PMC6868018 31799253

[14] Mo, Y., Wu, Y., Yang, X., Liu, F. & Liao, Y. Review the state-of-the-art technologies of semantic segmentation based on deep learning. *Neurocomputing* **493,** 626–646 (2022).

[15] Alsaafin, A., Nejat, P., Shafique, A., Khan, J., Alfasly, S., Alabtah, G. & Tizhoosh, H. R. SPLICE - Streamlining Digital Pathology Image Processing. *Am. J. Pathol.* (2024). 10.1016/j.ajpath.2024.06.007 39032601 10.1016/j.ajpath.2024.06.007

[16] Eccher, A., Pagni, F., Marletta, S., Munari, E. & Dei Tos, A. P. Perspective of a Pathologist on Benchmark Strategies for Artificial Intelligence Development in Organ Transplantation. *Crit. Rev. Oncog.* **28,** 1–6 (2023). 10.1615/CritRevOncog.2023048797 37968987

[17] L’Imperio, V., Brambilla, V., Cazzaniga, G., Ferrario, F., Nebuloni, M. & Pagni, F. Digital pathology for the routine diagnosis of renal diseases: a standard model. *J. Nephrol.* **34,** 681–688 (2021). 32683656 10.1007/s40620-020-00805-1 PMC8192318

[18] Sugawara, K. Training deep learning models for cell image segmentation with sparse annotations. *bioRxiv* 2023.06.13.544786 (2023). 10.1101/2023.06.13.544786

[19] Kirillov, A., Mintun, E., Ravi, N., Mao, H., Rolland, C., Gustafson, L., Xiao, T., Whitehead, S., Berg, A. C., Lo, W.-Y., Dollár, P. & Girshick, R. Segment Anything. *arXiv [cs.CV]* (2023). at <http://arxiv.org/abs/2304.02643>. Accessed 13 Dec 2023.

[20] Zhang, C., Han, D., Qiao, Y., Kim, J. U., Bae, S.-H., Lee, S. & Hong, C. S. Faster Segment Anything: Towards Lightweight SAM for Mobile Applications. *arXiv [cs.CV]* (2023). at <http://arxiv.org/abs/2306.14289>. Accessed 13 Dec 2023

[21] Bankhead, P., Loughrey, M. B., Fernández, J. A., Dombrowski, Y., McArt, D. G., Dunne, P. D., McQuaid, S., Gray, R. T., Murray, L. J., Coleman, H. G., James, J. A., Salto-Tellez, M. & Hamilton, P. W. QuPath: Open source software for digital pathology image analysis. *Sci. Rep.* **7,** 16878 (2017). 29203879 10.1038/s41598-017-17204-5 PMC5715110

[22] Altini, N., Rossini, M., Turkevi-Nagy, S., Pesce, F., Pontrelli, P., Prencipe, B., Berloco, F., Seshan, S., Gibier, J.-B., Pedraza Dorado, A., Bueno, G., Peruzzi, L., Rossi, M., Eccher, A., Li, F., Koumpis, A., Beyan, O., Barratt, J., Vo, H. Q., Mohan, C., Van Nguyen, H., Cicalese, P. A., Ernst, A., Gesualdo, L., Bevilacqua, V. & Becker, J. U. Performance and limitations of a supervised deep learning approach for the histopathological Oxford Classification of glomeruli with IgA nephropathy. *Comput. Methods Programs Biomed.* **242,** 107814 (2023). 37722311 10.1016/j.cmpb.2023.107814

[23] Wahab, N., Miligy, I. M., Dodd, K., Sahota, H., Toss, M., Lu, W., Jahanifar, M., Bilal, M., Graham, S., Park, Y., Hadjigeorghiou, G., Bhalerao, A., Lashen, A. G., Ibrahim, A. Y., Katayama, A., Ebili, H. O., Parkin, M., Sorell, T., Raza, S. E. A., Hero, E., Eldaly, H., Tsang, Y. W., Gopalakrishnan, K., Snead, D., Rakha, E., Rajpoot, N. & Minhas, F. Semantic annotation for computational pathology: multidisciplinary experience and best practice recommendations. *Hip Int.* **8,** 116–128 (2022). 10.1002/cjp2.256 PMC8822374 35014198

[24] Lu, M. Y., Williamson, D. F. K., Chen, T. Y., Chen, R. J., Barbieri, M. & Mahmood, F. Data-efficient and weakly supervised computational pathology on whole-slide images. *Nat Biomed Eng* **5,** 555–570 (2021). 33649564 10.1038/s41551-020-00682-w PMC8711640

[25] Caputo, A., L’Imperio, V., Merolla, F., Girolami, I., Leoni, E., Della Mea, V., Pagni, F. & Fraggetta, F. The slow-paced digital evolution of pathology: lights and shadows from a multifaceted board. *Pathologica* **115,** 127–136 (2023). 37387439 10.32074/1591-951X-868 PMC10462988

[26] Campanella, G., Hanna, M. G., Geneslaw, L., Miraflor, A., Werneck Krauss Silva, V., Busam, K. J., Brogi, E., Reuter, V. E., Klimstra, D. S. & Fuchs, T. J. Clinical-grade computational pathology using weakly supervised deep learning on whole slide images. *Nat. Med.* **25,** 1301–1309 (2019). 10.1038/s41591-019-0508-1 PMC7418463 31308507

[27] Krenzer, A., Makowski, K., Hekalo, A., Fitting, D., Troya, J., Zoller, W. G., Hann, A. & Puppe, F. Fast machine learning annotation in the medical domain: a semi-automated video annotation tool for gastroenterologists. *Biomed. Eng. Online* **21,** 33 (2022). 35614504 10.1186/s12938-022-01001-x PMC9134702

[28] Liu, K., Mitchell, K. J., Chapman, W. W. & Crowley, R. S. Automating tissue bank annotation from pathology reports - comparison to a gold standard expert annotation set. *AMIA Annu. Symp. Proc.* **2005,** 460–464 (2005). 16779082 PMC1560700

[29] Chen, C., Lu, M. Y., Williamson, D. F. K., Chen, T. Y., Schaumberg, A. J. & Mahmood, F. Fast and scalable search of whole-slide images via self-supervised deep learning. *Nat Biomed Eng* **6,** 1420–1434 (2022). 36217022 10.1038/s41551-022-00929-8 PMC9792371

[30] Saldanha, O. L., Muti, H. S., Grabsch, H. I., Langer, R., Dislich, B., Kohlruss, M., Keller, G., van Treeck, M., Hewitt, K. J., Kolbinger, F. R., Veldhuizen, G. P., Boor, P., Foersch, S., Truhn, D. & Kather, J. N. Direct prediction of genetic aberrations from pathology images in gastric cancer with swarm learning. *Gastric Cancer* **26,** 264–274 (2023). 36264524 10.1007/s10120-022-01347-0 PMC9950158

[31] Nakagawa, K., Moukheiber, L., Celi, L. A., Patel, M., Mahmood, F., Gondim, D., Hogarth, M. & Levenson, R. AI in Pathology: What could possibly go wrong? *Semin. Diagn. Pathol.* **40,** 100–108 (2023). 36882343 10.1053/j.semdp.2023.02.006 PMC13175311

[32] McKay, F., Williams, B. J., Prestwich, G., Bansal, D., Hallowell, N. & Treanor, D. The ethical challenges of artificial intelligence-driven digital pathology. *Hip Int.* **8,** 209–216 (2022). 10.1002/cjp2.263 PMC8977272 35174655

[33] Kim, I., Kang, K., Song, Y. & Kim, T.-J. Application of Artificial Intelligence in Pathology: Trends and Challenges. *Diagnostics (Basel)* **12,** (2022). 10.3390/diagnostics12112794 PMC9688959 36428854

[34] Selnes, O., Bjørsum-Meyer, T., Histace, A., Baatrup, G. & Koulaouzidis, A. Annotation Tools in Gastrointestinal Polyp Annotation. *Diagnostics (Basel)* **12,** (2022). 10.3390/diagnostics12102324 PMC9600922 36292013

[35] Gorman, C., Punzo, D., Octaviano, I., Pieper, S., Longabaugh, W. J. R., Clunie, D. A., Kikinis, R., Fedorov, A. Y. & Herrmann, M. D. Interoperable slide microscopy viewer and annotation tool for imaging data science and computational pathology. *Nat. Commun.* **14,** 1572 (2023). 36949078 10.1038/s41467-023-37224-2 PMC10033920

[36] Alcaraz-Mateos, E., Hernández-Gómez, R., Rojas Calvente, E., Sánchez-Campoy, N., Martínez González-Moro, I., Caballero-Alemán, F. & Poblet, E. Comparison of muscle activity while using different input devices in digital pathology. *Rev. Esp. Patol.* **55,** 19–25 (2022). 10.1016/j.patol.2021.02.005 34980436

[37] Molin, J., Lundström, C. & Fjeld, M. A comparative study of input devices for digital slide navigation. *J. Pathol. Inform.* **6,** 7 (2015). 25774318 10.4103/2153-3539.151894 PMC4355836

[38] Mazurowski, M. A., Dong, H., Gu, H., Yang, J., Konz, N. & Zhang, Y. Segment anything model for medical image analysis: An experimental study. *Med. Image Anal.* **89,** 102918 (2023). 37595404 10.1016/j.media.2023.102918 PMC10528428

[39] Chauveau, B. & Merville, P. Segment Anything by Meta as a foundation model for image segmentation: a new era for histopathological images. *Pathology* **55,** 1017–1020 (2023). 37813761 10.1016/j.pathol.2023.09.003

[40] Zhang, J., Ma, K., Kapse, S., Saltz, J., Vakalopoulou, M., Prasanna, P. & Samaras, D. SAM-Path: A Segment Anything Model for Semantic Segmentation in Digital Pathology. *arXiv [eess.IV]* (2023). at <http://arxiv.org/abs/2307.09570>. Accessed 13 Dec 2023

[41] Miao, R., Toth, R., Zhou, Y., Madabhushi, A. & Janowczyk, A. Quick Annotator: an open-source digital pathology based rapid image annotation tool. *Hip Int.* **7,** 542–547 (2021). 10.1002/cjp2.229 PMC8503896 34288586

[42] Meirelles, A. L., Kurc, T., Saltz, J. & Teodoro, G. Effective active learning in digital pathology: A case study in tumor infiltrating lymphocytes. *Comput. Methods Programs Biomed.* **220,** 106828 (2022). 35500506 10.1016/j.cmpb.2022.106828

[43] Girolami, I., Pantanowitz, L., Marletta, S., Hermsen, M., van der Laak, J., Munari, E., Furian, L., Vistoli, F., Zaza, G., Cardillo, M., Gesualdo, L., Gambaro, G. & Eccher, A. Artificial intelligence applications for pre-implantation kidney biopsy pathology practice: a systematic review. *J. Nephrol.* **35,** 1801–1808 (2022). 35441256 10.1007/s40620-022-01327-8 PMC9458558

[44] Abel, J. T., Ouillette, P., Williams, C. L., Blau, J., Cheng, J., Yao, K., Lee, W. Y., Cornish, T. C., Balis, U. G. J. & McClintock, D. S. Display Characteristics and Their Impact on Digital Pathology: A Current Review of Pathologists’ Future ‘Microscope’. *J. Pathol. Inform.* **11,** 23 (2020). 33042602 10.4103/jpi.jpi_38_20 PMC7518209

[45] Clarke, E. L., Munnings, C., Williams, B., Brettle, D. & Treanor, D. Display evaluation for primary diagnosis using digital pathology. *J Med Imaging (Bellingham)* **7,** 027501 (2020). 10.1117/1.JMI.7.2.027501 PMC7177184 32341938

[46] Cazzaniga, G., Mascadri, F., Marletta, S., Caputo, A., Guidi, G., Gambaro, G., Eccher, A., Dei Tos, A. P., Pagni, F. & L’Imperio, V. Benchmarking digital displays (monitors) for histological diagnoses: the nephropathology use case. *J. Clin. Pathol.* (2024). 10.1136/jcp-2024-209418 38538076 10.1136/jcp-2024-209418 PMC12573376

