# Supplementary figures and images for: Improving the Annotation Process in Computational Pathology: A Pilot Study with Manual and Semi-automated Approaches on Consumer and Medical Grade Devices
Source: J Imaging Inform Med. 2024 Sep 4;38(2):1112–9. doi: 10.1007/s10278-024-01248-x (PMC11950598; doi:10.1007/s10278-024-01248-x)

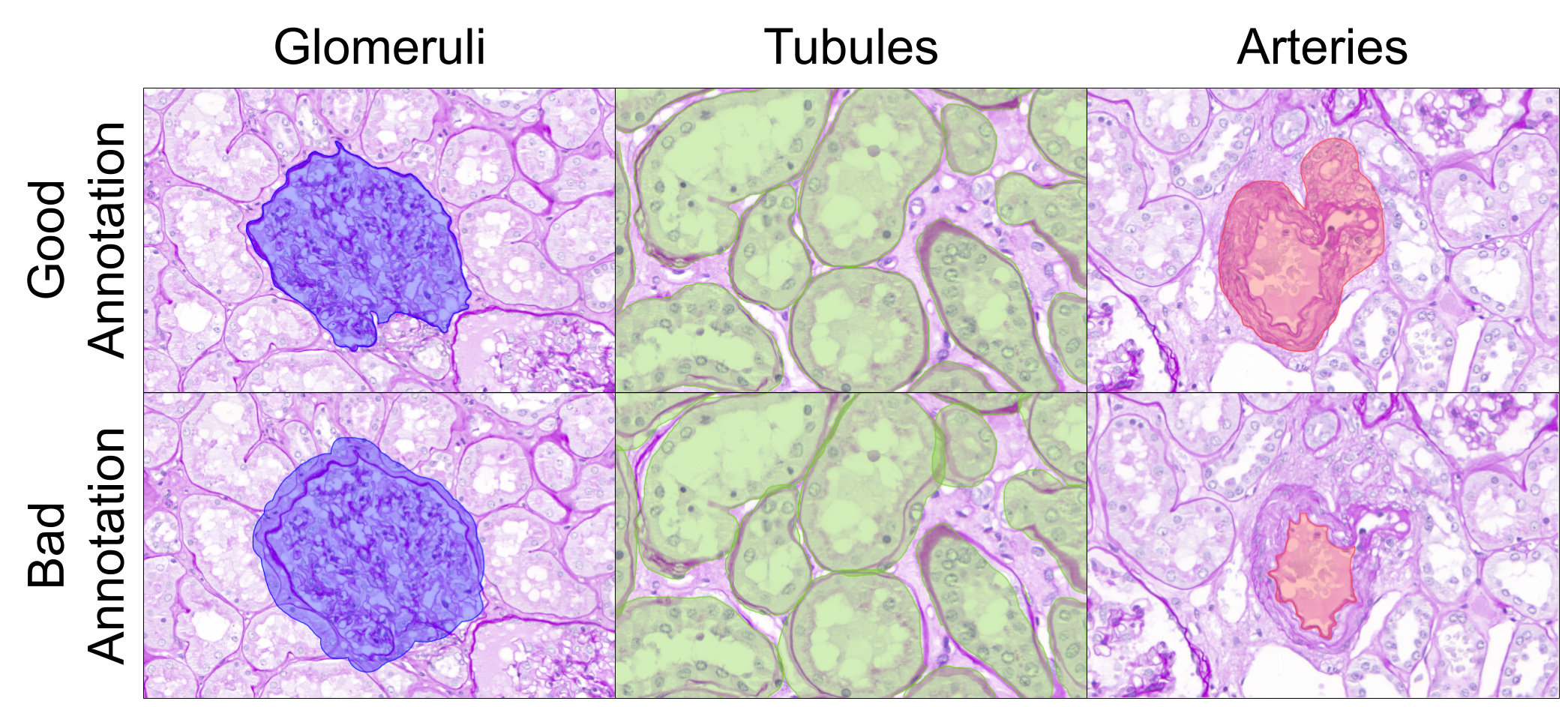

Supplement: Supplementary file 1 — Supplementary file1 : Examples of annotations with high and low accuracy. In the upper row, the Bowman’s capsule and the basal membrane of the tubules are followed precisely, with no inclusion of interstitial tissue nor exclusion of areas that are part of the structures of interest; the artery is fully included, encompassing all layers: intima, media, and adventitia. In the lower row, however, the membranes are not followed precisely, and only the intima layer and the lumen of the vessel are included, as SAM has identified the boundaries with high contrast of the elastic lamina compared to the more blurred contours between the adventitia and the interstitium (PNG 4047 KB) [file 10278_2024_1248_MOESM1_ESM.png]
