# Supplementary material for: Improving the Annotation Process in Computational Pathology: A Pilot Study with Manual and Semi-automated Approaches on Consumer and Medical Grade Devices
Source: J Imaging Inform Med. 2024 Sep 4;38(2):1112–9. doi: 10.1007/s10278-024-01248-x (PMC11950598; doi:10.1007/s10278-024-01248-x)
Supplement: Supplementary file 2 — Supplementary file2 (DOCX 9 KB) [file 10278_2024_1248_MOESM2_ESM.docx]

**Supplementary Methods**

*Annotation details, training and definitions*

WSI from the renal cortex was scanned using a MIDI II scanner (3D HISTECH, Budapest, Hungary). Both operators were provided with predefined rectangular regions of interest (ROIs) on the slide and reference images clearly indicating the structures to annotate within each ROI. Specifically, the first ROI involved the semantic annotation of tubules, well-delimited back-to-back structures, glomeruli, well-delimited separated structures, and arteries, difficult-to-identify, separated structures. For the definitions of the different structures to be annotated, the indications already provided in previous reports were used[^1^](https://paperpile.com/c/1aAFSI/ACpP). Before the annotation process started, the pathologist at its beginner phase in the field was trained on the recognition of the different structures and a preliminary session of annotation was performed with the experienced pathologist before starting with those of the study.

*Annotation devices settings*

Both operators utilized the mouse with standard controls (left and right clicks, mouse wheel) for annotation, employing the brush tool and keyboard shortcuts for efficiency. For the pad, a setup was chosen to swiftly switch between the brush and move tools, utilizing multi-finger gestures for zooming and navigating the slides. The pad used is specifically designed to work seamlessly with the BARCO MDPC-8127 monitor. The SAM technique varied across ROIs: for the first ROI containing tubules, the 'Auto mask' tool was preferred, whereas for the second and third ROIs, which included glomeruli and arteries, the operators favored using a prompt with the rectangle tool for bounding box annotations.

*Reproducibility metrics*

The overlap fraction (ov) typically refers to the ratio of the number of overlapping pixels between the predicted and ground truth regions to the total number of annotated pixels in the ground truth region. The Intersection over Union (IoU), on the other hand, is calculated as the ratio of the area of overlap between the predicted and ground truth regions to the total area encompassed by their union. IoU may vary if the background is large because it considers both the overlapping and non-overlapping areas within the combined regions. In cases with a significant background, small discrepancies in the predicted region relative to the ground truth can lead to a proportionally larger reduction in IoU, as the union area becomes much larger than the overlap area, thus reducing the IoU value.

1. [Bouteldja, N., Klinkhammer, B.M., Bülow, R.D., Droste, P., Otten, S.W., Freifrau von Stillfried, S., Moellmann, J., Sheehan, S.M., Korstanje, R., Menzel, S., et al. (2021). Deep Learning-Based Segmentation and Quantification in Experimental Kidney Histopathology. J. Am. Soc. Nephrol. *32*, 52–68.](http://paperpile.com/b/1aAFSI/ACpP) [10.1681/ASN.2020050597](http://dx.doi.org/10.1681/ASN.2020050597)[.](http://paperpile.com/b/1aAFSI/ACpP)
